# Correlation of Apobec Mrna Expression with overall Survival and pd-l1 Expression in Urothelial Carcinoma

**DOI:** 10.1038/srep27702

**Published:** 2016-06-10

**Authors:** Stephanie A. Mullane, Lillian Werner, Jonathan Rosenberg, Sabina Signoretti, Marcella Callea, Toni K. Choueiri, Gordon J. Freeman, Joaquim Bellmunt

**Affiliations:** 1 Bladder Cancer Center, Dana-Farber Cancer Institute/Brigham and Women’s Hospital, Boston MA, USA; 2 Biostatistics and Computational Biology, Dana-Farber Cancer Institute, Boston, MA, USA; 3 Memorial Sloan Kettering Cancer Center, New York, NY, USA; 4 Department of Pathology, Brigham and Women’s Hospital, Boston, MA, USA; 5 Harvard Medical School, Boston, MA, USA; 6 Department of Medical Oncology, Dana-Farber Cancer Institute, Boston, MA, USA; 7 University Hospital del Mar-IMIM, Barcelona, Spain.

## Abstract

Metastatic urothelial carcinoma (mUC) has a very high mutational rate and is associated with an APOBEC mutation signature. We examined the correlation of APOBEC expression with overall survival (OS) and PD-L1 expression in a cohort of 73 mUC patients. mRNA expression of APOBEC3 family of genes (A3A, A3B, A3C, A3F_a, A3F_b, A3G, A3H) was measured using Nanostring. PD-L1 expression, evaluated by immunohistochemistry, on tumor infiltrating mononuclear cells (TIMCs) and tumor cells was scored from 0 to 4, with 2–4 being positive. Wilcoxon’s non-parametric tests assessed the association of APOBEC and PD-L1. The Cox regression model assessed the association of APOBEC with OS. All APOBEC genes were expressed in mUC. Increased A3A, A3D, and A3H expression associates with PD-L1 positive TIMCs (p = 0.0009, 0.009, 0.06). Decreased A3B expression was marginally associated with PD-L1 positive TIMCs expression (p = 0.05). Increased A3F_a and A3F_b expression was associated with increased expression of PD-L1 on tumor cells (p = 0.05). Increased expression of A3D and A3H was associated with longer OS (p = 0.0009). Specific APOBEC genes have different effects on mUC in terms of survival and PD-L1 expression. A3D and A3H may have the most important role in mUC as they are associated with OS and PD-L1 TIMC expression.

In the United States, there were more than 76,000 cases and more than 16,000 deaths from urothelial carcinoma (UC) in 2014^1^. Cisplatin-based chemotherapy has improved clinical outcomes in metastatic UC (mUC), nonetheless the median overall survival is only 14 to 15 months, and mUC mostly remains an incurable disease^2^.

APOBEC deaminase enzyme family normally creates predicable mutations in viral DNA, limiting the replication ability of transposons and viruses^3^,^4^,^5^,^6^. Recently, APOBEC3 family has been shown to be a major source of somatic driver and passenger mutations in cancer^7^,^8^. The APOBEC3 family consists of seven members; APOBEC3A (A3A), APOBEC3B(A3B), APOBEC3C (A3C), APOBEC3D (A3D), APOBEC3F_a(A3F_a), APOBEC3F_b (A3F_b), APOBEC3G (A3G), and APOBEC3H (A3H).

Urothelial cancer has one of the highest mutational rates of all cancers, mean of 7.7 mutations per megabase^7^,^9^. The mechanism of this high mutation rate in UC is unknown. Although smoking has an epidemiologic association with UC, smoking carcinogens are not the cause of the majority of mutations based on mutational clustering analyses^7^,^10^,^11^. Mutational clustering observed in UC TCGA specimens were predominately TCW -> TTW or TGW changes, consistent with mutations caused by the APOBEC family of cytidine deaminases^7^,^10^,^12^,^13^,^14^. Also suggestive of APOBEC activity in UC was the high expression of APOBEC3B in almost all UC TCGA specimens^7^.

Multiple studies have demonstrated a correlation between A3B and A3A overexpression with mutational load, induction of DNA damage markers, and cell death^15^,^16^,^17^. It has been demonstrated that increased number of mutations correlate with better response rates to chemotherapy^18^. We hypothesized that increased APOBEC expression, which potentially plays a causative role in UC, would increase the mutation rate, thus increasing chemotherapy efficacy and OS as well as increasing PD-L1 positivity and increasing response to immunotherapy.

Assuming that increased APOBEC expression would cause more mutations, we inferred that increased expression of APOBEC correlates with PD-L1(CD274, B7-H1) expression in both tumor and immune cells. A higher mutational burden has been shown to lead to a higher neoantigen load. Since the anti-tumor immune response targets neoantigens, a higher neoantigen load means more reactive T cells and IFN-g production^19^. As a tumor evolves to evade the immune response, this IFN-g production may increase PD-L1 expression on tumor cells and TIMC, strengthening immune evasion. This could help to explain response to immunotherapy and will deserve further exploration in immunotherapy treated patients in future trials.

In a clinically annotated cohort of metastatic bladder cancer patients treated with platinum based therapy, we analyzed the association of APOBEC mRNA expression with PD-L1 expression, and overall survival (OS).

## Methods

### Patients and samples

73 mUC patients were identified from Brigham and Women’s Hospital and Hospital del Mar in Barcelona (Spain). Formalin fixed paraffin-embedded (FFPE) specimens from radical cystectomy or transurethral resection of bladder tumors were retrieved from the departments of pathology. All patients subsequently developed metastatic disease and received platinum based first line therapy. Prognostic factors including ECOG PS at initiation of chemotherapy, and whether patients had visceral site of metastasis, and clinical follow up data were collected. All subjects provided written informed consent. Institutional Review Board approval was obtained at Hospital del Mar and Dana-Farber/Harvard Cancer Centerbefore data acquisition and tumor staining, and all research was performed in accordance with the approved guidelines.

### mRNA expression and mutational analysis

mRNA expression profile of 300 genes, chosen based on their known or potential role in UC, was measured using Nanostring technology. Oligonucleotide probes for all genes analyzed were synthesized by Nanostring, and transcripts were counted using the automated Nanostring nCounter system. Counts were normalized with the nSolver Analysis Software (v1.0) in which mRNA expression was compared to internal Nanostring controls, several housekeeping genes, and invariant genes in bladder cancer identified by analyzing gene expression variances in several published datasets^20^,^21^,^22^. For this analysis, we only looked at APOBEC mRNA expression. Throughput mutation profiling was performed by using both mass spectroscopy-based genotyping (Oncomap 3 platform) and confirmed with hME sequencing (Supplementary Table 1).

### Immunohistochemistry and scoring of PD-L1 Expression

A tissue micro array (TMA) was constructed from treatment-naïve primary UC tissue. PD-L1 expression was evaluated by immunohistochemistry using a mouse monoclonal anti-PD-L1 antibody (405.9A11) developed in Dr. Gordon Freeman’s laboratory (Dana-Farber Cancer Institute, Boston, MA)^23^,^24^,^25^ Tumor-infiltrating mononuclear cells (TIMCs), PD-L1 expression on tumor cells and TIMCs with membranous expression was determined by two independent pathologists (MC, SS). PD-L1 tumor positivity was defined as ≥5% of tumor cell membrane staining. The extent of TIMCs was assessed in hematoxylin and eosin-stained slides and recorded as absent (0), focal (1), mild (2), moderate (3) and high (4) with score 0 or 1 considered negative. The extent of PD-L1-positive TIMCs was also assessed using the same scoring scale (0–4) and samples with a score of 2–4 were considered PD-L1 positive. For additional information, please see ref. 25.

### Statistical Analysis

Overall survival (OS) was defined from the start of first line chemotherapy to the date of death or censored on the last known date alive. Cox regression model was used to assess the association of APOBEC expression with OS in multivariable analysis adjusting for ECOG status and whether patients had visceral disease. Hazard ratio and 95% CI are also listed. Wilcoxon’s non-parametric tests were used to summarize the associations of expression of APOBEC genes, and PD-L1 expression on TIMCs and tumor cells. Hotspot mutations correlation with APOBEC gene expression was assessed using Wilcoxon’s non-parametric test treating gene expression as continuous variables. All statistical analyses were performed using SAS 9.4 (SAS Institute, NC). All tests were two-sided and a p-value of <0.05 was considered statistically significant.

## Results

Patient characteristics (n = 73) are presented in Table 1. All patients were included in the phase I clinical trial of cisplatin, gemcitabine, and paclitaxel (TCG) or phase II clinical trial comparing TCG vs. GC^26^.Median OS is 13 months and 41 patients died at time of data collection. Median follow up is 21 months.

PD-L1 tumor cell and PD-L1 MNC expression across the entire cohort is described in Table 2. mRNA expression level of APOBEC3A (A3A), APOBEC3B (A3B), APOBEC3C (A3C), APOBEC3D (A3D), APOBEC3F_a (A3F_a), APOBEC3F_b (A3F_b), APOBEC3G (A3G), and APOBEC3H (A3H) were measured using Nanostring and expression levels were dichotomized at the median. Median and quartile values are presented in Table 3.

We initially explored the correlation between APOBEC expression and OS, as increased mutations correlate with response to platinum in UC^18^ (Table 4). High expression of A3A, A3D, and A3H were correlated with longer OS in multivariate analysis (p = 0.01 [HR:0.45 (0.23–0.85)], p = 0.02 [HR = 0.46 (0.24, 0.88), p = 0.004 [HR = 0.38(0.19, 0.73)], respectively).

Subsequently, we investigated the association between APOBEC3 family expression and PD-L1 positivity in tumor cells and TIMCs, as previous evidence indicated specific members of the APOBEC3 family increases the number of mutations, and an increased mutational burden was associated with increased positive PD-L1 staining^27^.

**Figure 1:**
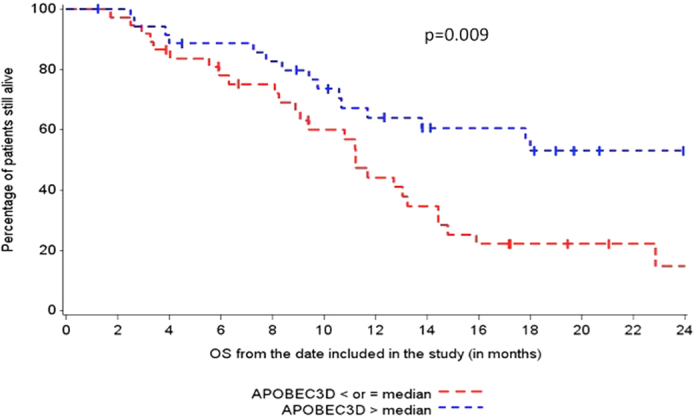
Association of APOBEC3D and OS.

**Figure 2:**
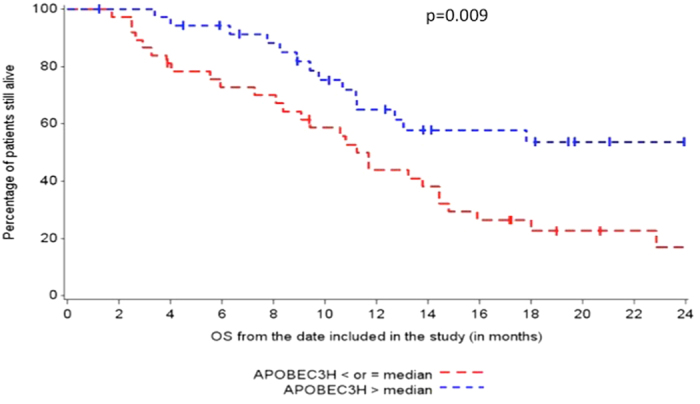
Association of APOBEC3H and OS.

Increased expression of A3A and A3D were significantly correlated with presences of TIMCs (p = 0.007, p = 0.05, respectfully) (Table 3).Increased expression of A3A, A3D, and A3H was associated with increased expression of PD-L1 on TIMCs (p = 0.0009, 0.0009, 0.06, respectively)_(Figs 1 and 2). Decreased expression of A3B was marginally associated with increased expression of PD-L1 on TIMCs (p = 0.05) (Table 4).

While PD-L1 expression on TIMCs has been associated with OS and an improved response to the checkpoint inhibitors in mUC^28^,^29^,^30^,^31^, we also analyzed the association between PD-L1 expression on tumor cells and APOBEC expression, as it may provide insight about how the expression of these two proteins within the same tumor affect each other. While increased expression of A3A, A3D, and A3H correlated with increased PD-L1 expression in TIMCs, low expression of A3F_a and A3F_b was associated with increased expression of PD-L1 on tumor cells (p = 0.04). No other APOBEC gene expression was associated with PD-L1 expression in tumor cells.

We summarized number of hotspot mutations, using Oncomapv3 (Table 4) in a subset of patients. We grouped number of mutations as 0 vs. 1(Supplementary Table 2). There was no significant association found between having hotspot mutations in these select genes and APOBEC gene expression (p-values-A3A: 0.09, A3B: 0.80, A3C: 0.79, A3D: 0.21, A3F: 0.64, A3G: 0.39, A3H: 0.20).

## Discussion

Many cancers are triggered by genomic instability. Instability can be induced by external factors, including UV, carcinogens, or smoking, or it can be induced by internal factors including mutations in MSH1 and BRCA1^32^,^33^ genes. Recently, evidence has emerged that the APOBEC family promotes genomic instability in cancer by causing specific mutations in tumors^10^,^13^,^16^,^34^,^35^. UC has one of the highest mutational rates of all cancers in the TCGA analysis^7^,^9^. It is predicted, based on mutational clustering, that the high mutation rate in UC is largely caused by the APOBEC enzyme family^10^.

In this study, we examined the association between APOBEC expression with OS and PD-L1 expression on TIMCs and tumor cells. Increased expression of A3A and A3D were correlated with increased TIMC presence. All of these values became more significant when correlating APOBEC expression with increased PD-L1 expression in TIMCs, indicating APOBEC expression may increase TIMC presence along PD-L1 expression. We also saw decreased expression of A3B with increased PD-L1 expression in TIMCs.

A significant association between A3A, A3D, and A3H with OS was observed, which might be driven by these APOBECs causing a high mutational burden. A3D and A3H both affect cell cycle regulation^6^. A3H is small, nuclear bound and interacts with DNA in interphase and telophase, whereas A3D is known to cause cell cycle profile changes in HIV^36^. These APOBEC enzymes may cause a high mutational burden and might be responsible for chemotherapy^18^ response leading to survival benefit in a similar way to what is seen with immunotherapy^27^.

We observed the strongest association between A3A and PD-L1 expression. Higher expression of A3A was also correlated with TIMC presence and longer OS. Recently Chan et al described that, based on the mutational pattern, A3A is more likely than A3B to be responsible for the majority of mutations in UC and other tumor types^7^,^34^, potentially explaining the strong association we observed. Overexpression of A3A is also known to cause more mutations than other APOBEC enzymes^34^, whereas A3B was the highest expressed APOBEC gene in the TCGA analysis and is also correlated with increased mutations in breast and UC^7^,^37^. In our study, A3B expression was higher than A3A, indicating that the expression level in which APOBEC enzymes produce mutation may be different for the different enzymes.

Decreased expression of A3F_a and A3F_b were associated with increased PD-L1 expression in tumor cells. It is known that A3F lacks efficacy in causing mutations, as demonstrated by work done in HIV^38^,^39^, however it is unknown why we observed this opposite correlation. It is also possible that A3F and other APOBEC genes cause additional changes in the cell immune response, such as up regulating other immune checkpoint inhibitors, which down regulate PD-L1 expression.

Increased expression of A3G and A3C did not associate with increased PD-L1 expression in TIMCs, tumor cells, or OS. A3G has a different binding motif and is thought to not act in the same fashion as other APOBECs^10^. While A3C had the highest expression in our analysis, it has been shown to not be as potent at creating mutations compared to the other APOBEC genes^40^. Based on our observations, these genes might not play a strong role in UC mutagenesis.

Recently, immune checkpoint inhibitors, anti-PD1 and anti-PD-L1, have emerged as promising treatment strategies in UC^41^. Increased PD-L1 expression on tumor infiltrating immune cells is correlated with improved response to checkpoint inhibitors and with potentially better OS in mUC^24^,^41^. However, presently, there are no well-established predictive response biomarkers^42^ to either chemotherapy or checkpoint inhibitors. Several hypotheses have been presented to describe why some diseases are more responsive to these agents and why only select patients respond. Response to anti-PD-L1 therapy has been correlated with PD-L1 expression in tumor and immune cells, alterations in PIK3/AKT pathway, STAT3/JAK3 pathway, specific neoantigens expression, and mutational load^27^,^43^,^44^,^45^,^46^,^47^. In UC, it is hypothesized that responses to checkpoint inhibitors is due to the high mutational rate or frequent alterations in the PIK3/AKT pathway^48^,^49^,^50^. We can hypothesize based on our findings, that APOBEC expression may be a predictor of response to immunotherapy, due to the likely increase in mutation rate^27^,^45^,^51^, however this needs to be confirmed in further studies.

There were multiple limitations to our study. First, we did not have next generation sequencing data on these samples, thus we had to assume the APOBEC expression is associated with the mutational signatures and burdens as has been previously reported^8^,^10^,^34^,^52^. The expression of APOBEC is likely driven by tumor cells, however single-cell sequencing will provide additional evidence to this hypothesis.

In a brief exploration of mass spectrometry based hot spot sequencing of these samples, hot spot mutation burden was not correlated with any APOBEC expression. Tissue sample analyzed were obtained from local tumors at the time of diagnosis and not from distant metastatic locations. It is known that PD-L1 expression and mutational burden can differ between primary and metastatic sites in other tumor types^25^,^53^. It is also unknown about how other clinical variables or treatment variables affect PDL1 expression. This may be different in different tumor types, like the differences seen between smoking and PD-L1 expression in bladder versus NSCLC^24^,^54^. There is considerably more research that needs to be completed. To confirm these results, a large prospective cohort of uniformly treated mUC patients, ideally, comparing primary and metastatic tissue. Our findings should be confirmed in chemotherapy treated patients and expanded into immunotherapy treated patients. If confirmed, APOBEC expression may be used to identify patients who respond well to metastatic chemotherapy. Of great interest would be to comparing APOBEC expression, APOBEC signatures, mutational load, and response to chemotherapy and immunotherapy in UC.

Overall, we observed increased expression of APOBEC genes that also associated with increased PD-L1 expression and OS. This may indicate that patients with increased expression of these proteins derive a survival benefit when receiving platinum based chemotherapy and we can hypothesize that they might be more likely to respond to checkpoint inhibitors. These observations require prospective validation and warrant future study in the ongoing checkpoint inhibitors trials in UC that are looking for better predictive factors of response.

## Additional Information

**How to cite this article**: Mullane, S. A. *et al*. Correlation of Apobec Mrna Expression with overall Survival and pd-l1 Expression in Urothelial Carcinoma. *Sci. Rep.* **6**, 27702; doi: 10.1038/srep27702 (2016).

## Supplementary Material

Supplementary Information

## Figures and Tables

**Table 1:** Patient Characteristics.

	N	%
ECOG PS		
0	24	33%
1	47	64%
2	2	3%
Visceral disease		
Without	42	58%
With	31	42%
Stage		
0	5	7%
1	5	7%
2	35	48%
3	23	32%
4	4	5%
Unknown	1	1%

**Table 2:** PD-L1 Expression.

	N	%
Mononuclear Cell presence (score = 2, 3, 4)		
0	1	1%
1	24	33%
2	21	29%
3	17	23%
4	3	4%
Unknown	7	10%
PD-L1 mononuclear presence (score = 2, 3, 4)		
0	19	26%
1	21	29%
2	18	25%
3	6	8%
4	2	3%
Unknown	7	10%
PD-L1 tumor (≥5%)		
Negative	63	86%
Positive	10	14%

**Table 3:** APOBEC expression.

Gene	Median Expression	Quartile 1 Expression	Quartile 3 Expression
APOBEC3A	14.09	6.79	26.27
APOBEC3B	43.13	23.37	83.28
APOBEC3C	162.04	100.51	264.17
APOBEC3D	59.48	44.61	97.2
APOBEC3F_b	67.91	50.43	98.51
APOBEC3F_a	30.51	19.31	51.87
APOBEC3G	97.03	71.81	163.87
APOBEC3H	13.26	7.37	21.79

**Table 4:** APOBEC overexpression correlation with PD-L1/PD-1 and OS.

Gene	MNC presence (score = 2, 3, 4)		PDL1 MNC presence (score = 2, 3, 4)		Longer OS			PDL1 tumor expression ( ≥ 5%)	
	P-vlaue		P-vlaue		HR, CI	P-vlaue		P-vlaue	
APOBEC3A	**0.007**	**High Expression**	**0.0009**	**High expression**	**0.45 (CI:0.23–0.86)**	**0.01**	**High expression**	0.87	
APOBEC3B	**0.13**		**0.05**	**Low expression**		0.89		0.18	
APOBEC3C	0.80		0.2			0.6		0.06	
APOBEC3D	**0.05**	**High Expression**	**0.009**	**High expression**	**0.49 (0.25–0.95)**	**0.02**	**High expression**	0.23	
APOCEC3F_a	0.40		0.49			0.43		**0.05**	**Low expression**
APOBEC3F_b	0.46		0.31			0.15		**0.04**	**Low expression**
APOBEC3G	0.59		0.65			0.55		0.47	
APOBEC3H	0.19		**0.06**	**High expression**	**0.36 (0.19–0.71)**	**0.004**	**High expression**	0.8	

Correlation of APOBEC expression with OS was performed using a Cox regression model in multivariate analysis adjusting for ECOG PS and visceral disease. Wilcoxon’s non-parametric tests were used to summarize the associations of expression of APOBEC genes, and PD-L1 expression on TIMCs and tumor cells. *HR: Hazard Ratio; *CI: Confidence Interval.
