# Supplementary material for: Correlation of Apobec Mrna Expression with overall Survival and pd-l1 Expression in Urothelial Carcinoma
Source: Sci Rep. 2016 Jun 10;6:27702. doi: 10.1038/srep27702 (PMC4901342; doi:10.1038/srep27702)
Supplement: Supplementary Information [file srep27702-s1.pdf]

Supplemental Information

CORRELATION of APOBEC mRNA EXPRESSION WITH OVERALL SURVIVAL AND PD-L1 EXPRESSION IN UROTHELIAL CARCINOMA

Stephanie A. Mullane, Lillian Werner, Jonathan Rosenberg, Sabina Signoretti, Marcella Callea, Toni K. Choueiri, Gordon J. Freeman, Joaquim Bellmunt

Supplementary Table 1

Gene List: ABL1, AKT1, ALK, APC, ATM, AURKC, BRAF, CDH1, CDKN2A, CTNNB1, DBN1, EGFR, EPHA3, EPHA4, EPHA5, EPHB1, ERBB2, FES, FGFR2, FGFR3, FLT3, HRAS, KIT, KRAS, LRP1B, MAP2K4, MSH6, MYCMYH1, NF2, NRAS, PDGFRA, PDGFRB, PIK3CA, PIK3R1, PKHD1, PTCH1, PTEN, PTPN11, RB1, ROBO2, SMAD4, SPTAN1, SRC, TP53, TSHR, VHL

Supplementary Table 2

	N	%
**Number of mutated genes**		
0	34	47%
1	19	26%
2	10	14%
3	5	7%
4 or more	5	7%
